# Elderly onset age is associated with low efficacy of first anti-tumor necrosis factor treatment in patients with inflammatory bowel disease

**DOI:** 10.1038/s41598-022-09455-8

**Published:** 2022-03-29

**Authors:** Takahiro Amano, Shinichiro Shinzaki, Akiko Asakura, Taku Tashiro, Mizuki Tani, Yuriko Otake, Takeo Yoshihara, Shuko Iwatani, Takuya Yamada, Yuko Sakakibara, Naoto Osugi, Shuji Ishii, Satoshi Egawa, Manabu Araki, Yuki Arimoto, Masanori Nakahara, Yoko Murayama, Ichizo Kobayashi, Kazuo Kinoshita, Hiroyuki Ogawa, Satoshi Hiyama, Narihiro Shibukawa, Masato Komori, Yorihide Okuda, Takashi Kizu, Shunsuke Yoshii, Yoshiki Tsujii, Yoshito Hayashi, Takahiro Inoue, Hideki Iijima, Tetsuo Takehara

**Affiliations:** 1 Department of Gastroenterology and Hepatology, Osaka University Graduate School of Medicine, 2-2 Yamadaoka, Suita, Osaka 565-0871 Japan; 2 Department of Gastroenterology and Hepatology, Osaka Rosai Hospital, Sakai, Osaka Japan; 3 Department of Gastroenterology, National Hospital Organization Osaka National Hospital, Osaka, Japan; 4 Department of Gastroenterology, Toyonaka Municipal Hospital, Toyonaka, Osaka Japan; 5 Department of Gastroenterology, Osaka General Medical Center, Osaka, Japan; 6 Department of Internal Medicine, Osaka Police Hospital, Osaka, Japan; 7 Department of Gastroenterology, National Hospital Organization Osaka Minami Medical Center, Kawachinagano, Osaka Japan; 8 Department of Gastroenterology, Kansai Rosai Hospital, Amagasaki, Hyogo Japan; 9 Department Gastroenterology, Ikeda City Hospital, Ikeda, Osaka Japan; 10 Department Gastroenterology and Hepatology, Itami City Hospital, Itami, Hyogo Japan; 11 Department of Gastroenterology, Higashiosaka City General Hospital, Higashiosaka, Osaka Japan; 12 Department of Gastroenterology, Otemae Hospital, Osaka, Japan; 13 Department of Gastroenterology, Nishinomiya Municipal Central Hospital, Nishinomiya, Hyogo Japan; 14 Department of Gastroenterology, Japan Community Healthcare Organization Osaka Hospital, Osaka, Japan; 15 Department of Gastroenterology, NTT-West Osaka Hospital, Osaka, Japan; 16 Department of Gastroenterology, Hyogo Prefectural Nishinomiya Hospital, Nishinomiya, Hyogo Japan; 17 Department of Gastroenterology, Saiseikai Senri Hospital, Suita, Osaka Japan; 18 Department of Gastroenterology, Yao Municipal Hospital, Yao, Osaka Japan

**Keywords:** Gastroenterology, Medical research

## Abstract

The outcomes of patients with elderly onset (EO) inflammatory bowel disease (IBD) treated with anti-tumor necrosis factor (TNF) remains uncertain. The present study evaluated the efficacy and safety of anti-TNF treatment for bio-naïve EO-IBD. Elderly patients were defined as those 60 years and older, and further divided into those with EO (Elderly-EO) and those with non-elderly onset (Elderly-NEO). A total of 432 bio-naïve patients were enrolled in this multicenter observational study, comprising 55 with Elderly-EO (12.7%), 25 with Elderly-NEO (5.8%), and 352 under age 60 (Non-elderly, 81.5%). After 52 weeks of anti-TNF treatment, clinical and steroid-free remission rates were significantly lower in Elderly-EO than in Non-elderly (37.7% and 60.8%; *P* = 0.001, and 35.9% and 57.8%; *P* = 0.003, respectively), and comparable between Elderly-NEO and Non-elderly. Multivariate analysis revealed that elderly onset was a significant factor for both clinical remission (OR, 0.49, 95% CI 0.25–0.96) and steroid-free remission (OR, 0.51, 95% CI 0.26–0.99) after 52 weeks of anti-TNF treatment. The rate of cumulative severe adverse events was significantly higher in Elderly-EO than in Non-elderly (*P* = 0.007), and comparable between Elderly-NEO and Non-elderly. In conclusion, anti-TNF treatment for bio-naïve EO-IBD may be less effective and raise safety concerns.

## Introduction

Inflammatory bowel disease (IBD), comprising ulcerative colitis (UC) and Crohn’s disease (CD), is a chronic inflammation in the intestinal tract that repeatedly relapses. The etiology of IBD is unclear but involves a complex interplay of genetic factors, environmental factors, gut microbiota alterations, and dysregulation of the host immune system^1–4^. These chronic diseases are most prevalent in the second and third decades of life. With the recent development of an aging society, however, IBD is presenting more in elderly patients, commonly defined as 60 years of age and older^1,5^. Approximately 10–15% of patients with IBD are diagnosed at age 60 or older (18.9 per 100,000 elderly individuals)^5–7^.

Elderly patients with IBD are classified as those with elderly onset (EO-IBD) and those with non-elderly onset (NEO-IBD) according to the timing of the disease onset and subsequent disease duration. In clinical practice, it is important to distinguish between elderly patients with EO-IBD and those with NEO-IBD because those with a longer disease duration have a lower frequency of hospitalization and IBD-related surgery, and a lower rate of corticosteroid use during disease exacerbation, especially patients with UC^8–10^. The disease phenotype also differs between EO-IBD and NEO-IBD^11,12^. Left-sided disease is most common among those with EO-UC^13^. In patients with EO-CD, colonic disease and inflammatory behavior are more common, whereas penetrating and perianal disease are less common than among those with NEO-CD^14^. Although EO-IBD is less aggressive and has a mild clinical course^5,11^, some studies recently suggested that EO-IBD, especially within 1 year of diagnosis, may also have an aggressive clinical course^15–17^. Furthermore, although prolonged corticosteroid therapy leads to a higher mortality rate than anti-tumor necrosis factor (TNF) therapy^18^, the cumulative use of corticosteroids, risk of IBD-related surgery, and mortality are similar between EO-IBD and NEO-IBD^16^, and those with EO-IBD are less likely to receive treatment with immunomodulators and anti-TNF agents compared to those with NEO-IBD^5,11,14–16,19^.

Anti-TNF treatment is effective for both the induction and maintenance of patients with IBD and improves their quality of life^20–22^. The efficacy and safety of anti-TNF treatment for EO-IBD are not yet clear, however, because elderly patients are generally not included in large-scale studies such as randomized controlled trials. Although a few observational cohorts have suggested that anti-TNF treatment for elderly patients with IBD, including those with NEO-IBD, have a higher rate of treatment discontinuation and severe adverse events such as infection^23–25^, there has been no comparative analysis investigating the outcomes of anti-TNF treatment by distinguishing between elderly patients with EO-IBD and NEO-IBD. This study, therefore, evaluated the efficacy and safety of anti-TNF treatment and investigated the factors associated with the effectiveness of anti-TNF treatment for bio-naïve EO-IBD.

## Results

### Patient characteristics

Among the 605 patients with IBD enrolled, 444 bio-naïve patients with IBD who underwent anti-TNF treatment were included in the study. Patients who underwent a total colectomy and/or colostomy before treatment were excluded from the analysis, and 432 bio-naïve patients with IBD were included in the analyses. Of the 432 bio-naïve patients included in the analysis, 217 (50.2%) had UC and 215 (49.8%) had CD; 55 (12.7%) patients were classified as Elderly-EO, 25 (5.8%) patients were classified as Elderly-NEO, and 352 (81.5%) were classified as Non-elderly. Baseline characteristics and concomitant drugs at the start of anti-TNF treatment are shown in Table 1. The proportion of patients with UC was significantly higher in the Elderly-NEO (68.0%) and Elderly-EO (80.0%) groups than in the Non-elderly group (44.3%, *P* = 0.021 or *P* < 0.001, respectively). Although disease duration was significantly longer in the Elderly-NEO group (15 years [95% CI 7–28]) than in the Non-elderly group (2 years [95% CI 0–9], *P* < 0.001), there was no significant difference between the Elderly-EO (1 year [95% CI 0–3]) and Non-elderly groups (*P* = 0.048). Regarding concomitant drugs, although the proportion of patients with concomitant corticosteroid and immunomodulators did not differ between the Elderly-NEO (32.0% and 40.0%) and Non-elderly groups (31.8% and 22.7%; *P* = 0.985 and *P* = 0.050, respectively), the proportions were higher in the Elderly-EO group (61.8% and 40.0%) than in the Non-elderly group (*P* < 0.001 and *P* = 0.006, respectively).

**Table 1:** Baseline characteristics and concomitant drugs at the start of anti-TNF treatment.

	Non-elderly	Elderly	
		Elderly-NEO	Elderly-EO
All, n (%)	352 (81.5)	25 (5.8)	55 (12.7)
Sex (male), n (%)	239 (67.9)	13 (52.0)	34 (61.8)
Age at the start of anti-TNF, median [IQR]	35 [23–45]	66 [63–70]*	69 [66–76]*
Observation period (months), median [IQR]	23 [7–54]	33 [7–57]	15 [3–47]*
Disease duration (years), median [IQR]	2 [0–9]	15 [7–28]*	1 [0–3]
Disease duration > 1 year, n (%)	223 (63.3)	25 (100.0)*	36 (65.5)
Current or past smoking, n (%)	92 (26.2)	8 (32.0)	18 (32.7)
**IBD type**			
UC, n (%)	156 (44.3)	17 (68.0)*	44 (80.0)*
Proctitis/Left-sided/Pancolitis, n	1/44/111	0/8/9	1/12/31
pMayo, median [IQR]	5 [4–7]	5 [3–8]	5 [3–7]
CD, n (%)	196 (55.4)	8 (32.0)*	11 (20.0)*
Ileal/Colonic/Ileocolonic, n	55/38/103	3/1/4	3/3/5
Non-stricturing, non-penetrating/Stricturing/Penetrating, n	61/96/39	1/5/2	4/7/0
Perianal disease, n (%)	91 (46.4)	3 (37.5)	0 (0.0)*
HBI, median [IQR]	5 [3–7]	7 [4–8]	6 [4–7]
Prior IBD surgery, n (%)	58 (29.6)	4 (50.0)	1 (9.1)
CRP < 0.30 mg/dl, n (%)	133 (38.0)	7 (28.0)	19 (34.6)
Alb < 3.6 g/dl, n (%)	168 (49.3)	17 (68.0)	33 (63.5)
**Anti-TNF agents**			
Infliximab, n (%)	203 (57.7)	17 (68.0)	34 (61.8)
Adalimumab, n (%)	137 (38.9)	4 (16.0)	17 (30.9)
Golimumab, n (%)	12 (3.4)	4 (16.0)*	16 (4.2)
**Concomitant drugs**			
5-ASA, n (%)	301 (85.5)	22 (88.0)	50 (90.9)
Corticosteroid, n (%)	112 (31.8)	8 (32.0)	34 (61.8)*
Immunomodulators, n (%)	80 (22.7)	10 (40.0)	22 (40.0)*

*Alb* Albumin, *anti-TNF* anti-tumor necrosis factor, *5-ASA* 5-aminosalicylate, *CD* Crohn’s disease, *CRP* C reactive protein, *EO* elderly onset, *HBI* Harvey-Bradshaw Index, *IBD* inflammatory bowel disease, *IQR* interquartile range, *NEO* non-elderly onset, *pMayo* partial Mayo score, *UC* ulcerative colitis, *P value < 0.025 compared with Non-elderly.

### Short- and long-term efficacy of anti-TNF treatment in Elderly-EO patients

**Figure 1:**
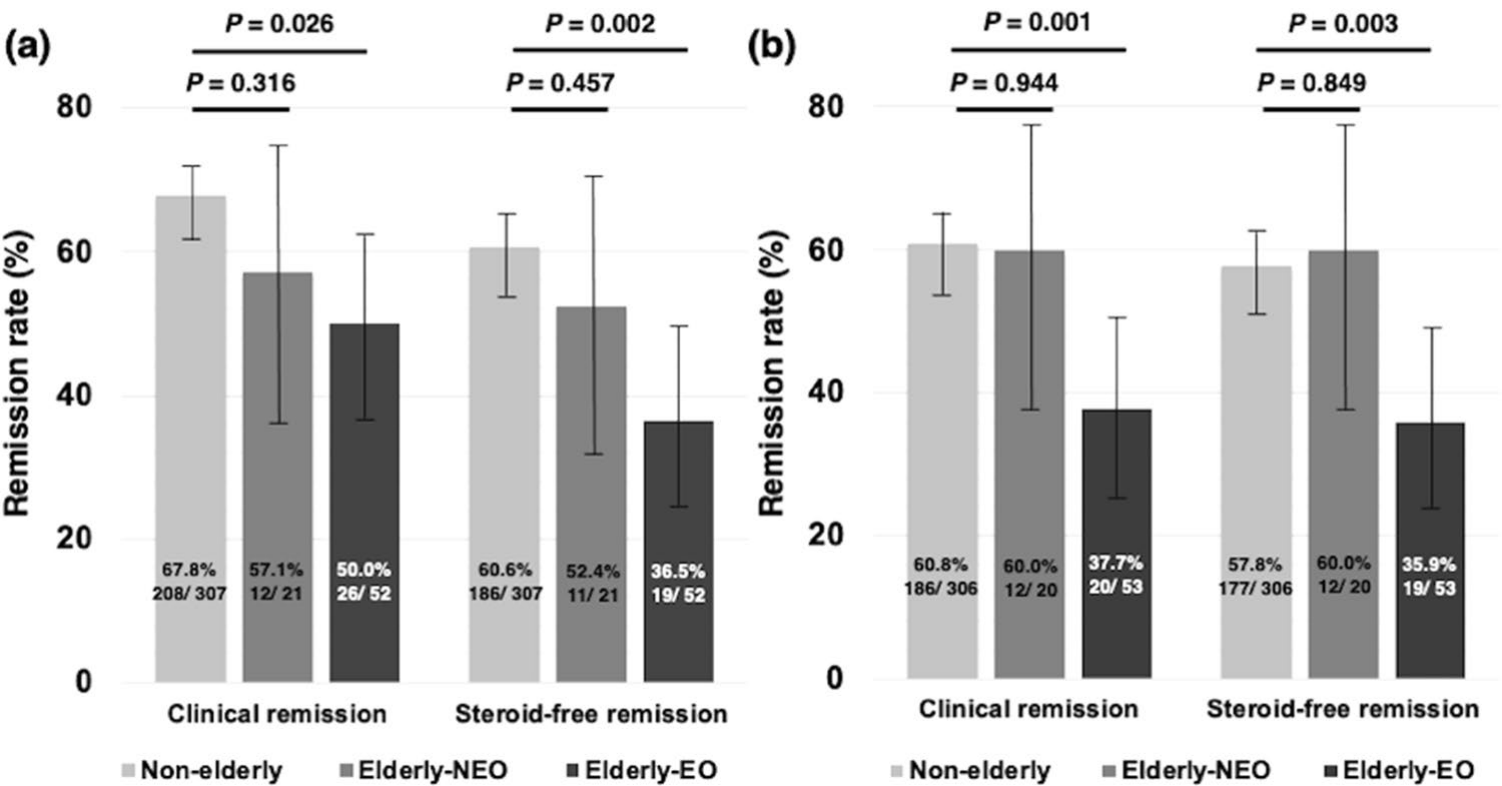
Efficacy of anti-TNF treatment for bio-naïve IBD patients. (**a**) Clinical and steroid-free remission after 8 weeks of anti-TNF treatment in Elderly-EO, Elderly-NEO, and Non-elderly groups. (**b**) Clinical and steroid-free remission after 52 weeks of anti-TNF treatment in Elderly-EO, Elderly-NEO, and Non-elderly groups. *Anti-TNF* anti-tumor necrosis factor, *EO* elderly onset, *IBD* inflammatory bowel disease, *NEO* non-elderly onset.

The clinical remission rate after 8 weeks of anti-TNF treatment tended to be lower in the Elderly-EO group (26/52, 50.0%, [95% CI 36.8–63.1]) than in the Non-elderly group (208/307, 67.8%, [95% CI 61.9–72.4], *P* = 0.026), and the steroid-free remission rate at 8 weeks of anti-TNF treatment was significantly lower in the Elderly-EO group (19/52, 36.5%, [95% CI 24.8–50.1]) than in the Non-elderly group (186/307, 60.6% [95% CI 54.6–65.5], *P* = 0.002; Fig. 1a). No significant difference was detected, however, between the Elderly-NEO (12/21, 57.1%, [95% CI 36.5–75.5] and 11/21, 52.4% [95% CI 32.3–71.6]) and Non-elderly groups (Fig. 1a). The clinical remission rate and steroid-free remission rate after 52 weeks of anti-TNF treatment were significantly lower in the Elderly-EO group (20/53, 37.7%, [95% CI 25.9–51.1] and 19/53, 35.9%, [95% CI 24.3–49.3]) than in the Non-elderly group (186/306, 60.8%, [95% CI 54.5–65.4] and 177/306, 57.8%, [95% CI 51.5–62.6], *P* = 0.001 and *P* = 0.003; Fig. 1b). No significant difference was detected, however, between the Elderly-NEO (12/20, 60.0% [95% CI 38.6–78.1] and 12/20, 60.0% [95% CI 38.6–78.1]) and Non-elderly groups (Fig. 1b).

### EO as a factor associated with the long-term efficacy of anti-TNF treatment

Next, we investigated the factors associated with clinical remission or steroid-free remission after 8 and 52 weeks of anti-TNF treatment in patients with IBD by multivariate analyses. At 8 weeks, a C-reactive protein (CRP) level of less than 0.30 mg/dl at week 0 was extracted as a significant factor for both clinical remission (OR, 1.93, 95% CI 1.10–3.39) and steroid-free remission (OR, 2.31, 95% CI, 1.30–4.08; Supplemental Table 1). Interestingly, after 52 weeks of anti-TNF treatment, EO (OR, 0.49, 95% CI 0.25–0.96) and UC (OR, 0.38, 95% CI 0.21–0.70) were extracted as a significant factor for clinical remission (Table 2). In addition, EO (OR, 0.51, 95% CI 0.26–0.99) and concomitant corticosteroid (OR, 0.51, 95% CI 0.29–0.89) were extracted as a significant factor for steroid-free remission (Table 2).

**Table 2:** Multivariate analysis of factors for clinical or steroid-free remission after 52 weeks of anti-TNF treatment (n = 379).

	Clinical remission		Steroid-free remission	
	OR (95% CI)	*P* value	OR (95% CI)	*P* value
Age at onset (EO)	0.49 (0.25–0.96)	0.040	0.51 (0.26–0.99)	0.048
Sex (male)	0.73 (0.45–1.18)	0.211	0.74 (0.46–1.20)	0.233
Disease duration > 1 year	0.85 (0.51–1.42)	0.548	0.81 (0.49–1.33)	0.410
Current or past smoking	0.88 (0.53–1.44)	0.614	0.78 (0.46–1.31)	0.353
IBD-type (UC)	0.38 (0.21–0.70)	0.001	0.62 (0.34–1.12)	0.114
CRP < 0.30 mg/dl	1.48 (0.89–2.47)	0.129	1.41 (0.85–2.34)	0.179
Alb < 3.6 g/dl	1.10 (0.67–1.80)	0.699	1.13 (0.69–1.85)	0.606
**Anti-TNF agents**				
Infliximab	1.0 (reference)		1.0 (reference)	
Adalimumab	1.15 (0.71–1.84)	0.556	1.09 (0.68–1.73)	0.712
Golimumab	1.33 (0.47–3.71)	0.582	1.43 (0.51–4.01)	0.490
**Concomitant drugs**				
Corticosteroid	0.95 (0.54–1.67)	0.869	0.51 (0.29–0.89)	0.018
Immunomodulators	0.91 (0.54–1.55)	0.869	1.01 (0.59–1.72)	0.963

*Alb* albumin, *Anti-TNF* anti-tumor necrosis factor, *CI* confidence interval, *CRP* C-reactive protein, *EO* elderly onset, *IBD* inflammatory bowel disease, *OR* odds ratio, *UC* ulcerative colitis.

### Characteristics and concomitant drugs associated with the long-term efficacy of anti-TNF treatment for Elderly-EO patients

We next explored the conditions in which anti-TNF treatment was more effective for Elderly-EO. Of 55 elderly-EO patients, clinical symptoms of 2 patients at 52 weeks after anti-TNF treatment could not be collected from medical records in our hospitals because those were transferred to the other hospitals until 52 weeks after anti-TNF treatment. In 53 Elderly-EO patients, we evaluated the characteristics or concomitant drugs associated with clinical remission or steroid-free remission after 52 weeks of anti-TNF treatment (Table 3). Of the 53 Elderly-EO patients, only 19 achieved clinical remission or steroid-free remission after 52 weeks of anti-TNF treatment. Therefore, a univariate analysis, not a multivariate analysis, was performed. Univariate analysis revealed that a disease duration greater than 1 year (OR, 0.26, 95% CI 0.07–0.85), IBD-type (UC; OR, 0.15, 95% CI 0.03–0.66), and concomitant corticosteroid therapy (OR, 0.25, 95% CI 0.07–0.81) were significant factors for clinical remission after 52 weeks of anti-TNF treatment (Table 3)**.** Furthermore, univariate analysis revealed that a disease duration greater than 1 year (OR, 0.22, 95% CI 0.06–0.74), IBD-type (UC) (OR, 0.13, 95% CI 0.02–0.59), and concomitant corticosteroid therapy (OR, 0.21, 95% CI 0.06–0.69) were significant factors for steroid-free remission after 52 weeks of anti-TNF treatment (Table 3).

**Table 3:** Univariate analysis of factors for clinical or steroid-free remission after 52 weeks of anti-TNF treatment in Elderly-EO (n = 53).

Variables	Clinical remission		Steroid-free remission	
	OR (95% CI)	*P* value	OR (95% CI)	*P* value
Sex (male)	1.94 (0.59–6.30)	0.267	1.71 (0.52–5.57)	0.372
Disease duration > 1 year	0.26 (0.07–0.85)	0.027	0.22 (0.06–0.74)	0.015
Current or past smoking	2.55 (0.77–8.38)	0.121	2.02 (0.61–6.62)	0.245
IBD-type (UC)	0.15 (0.03–0.66)	0.012	0.13 (0.02–0.59)	0.008
CRP < 0.30 mg/dl	0.94 (0.29–3.00)	0.920	0.74 (0.22–2.44)	0.628
Alb < 3.6 g/dl	0.82 (0.24–2.72)	0.749	1.04 (0.30–3.55)	0.940
**Anti-TNF agents**				
Infliximab	1.0 (reference)		1.0 (reference)	
Adalimumab	0.69 (0.19–2.48)	0.580	0.51 (0.13–1.93)	0.324
Golimumab	1.53 (0.19–12.2)	0.684	1.53 (0.19–12.3)	0.684
**Concomitant drugs**				
Corticosteroid	0.25 (0.07–0.81)	0.021	0.21 (0.06–0.69)	0.011
Immunomodulators	0.31 (0.09–1.06)	0.062	0.35 (0.10–1.21)	0.098

*Alb* albumin, *Anti-TNF* anti-tumor necrosis factor, *CI* confidence interval, *CRP* C-reactive protein, *EO* elderly onset, *IBD* inflammatory bowel disease, *OR* odds ratio, *UC* ulcerative colitis.

### Safety of anti-TNF treatment for Elderly-EO patients

**Figure 2:**
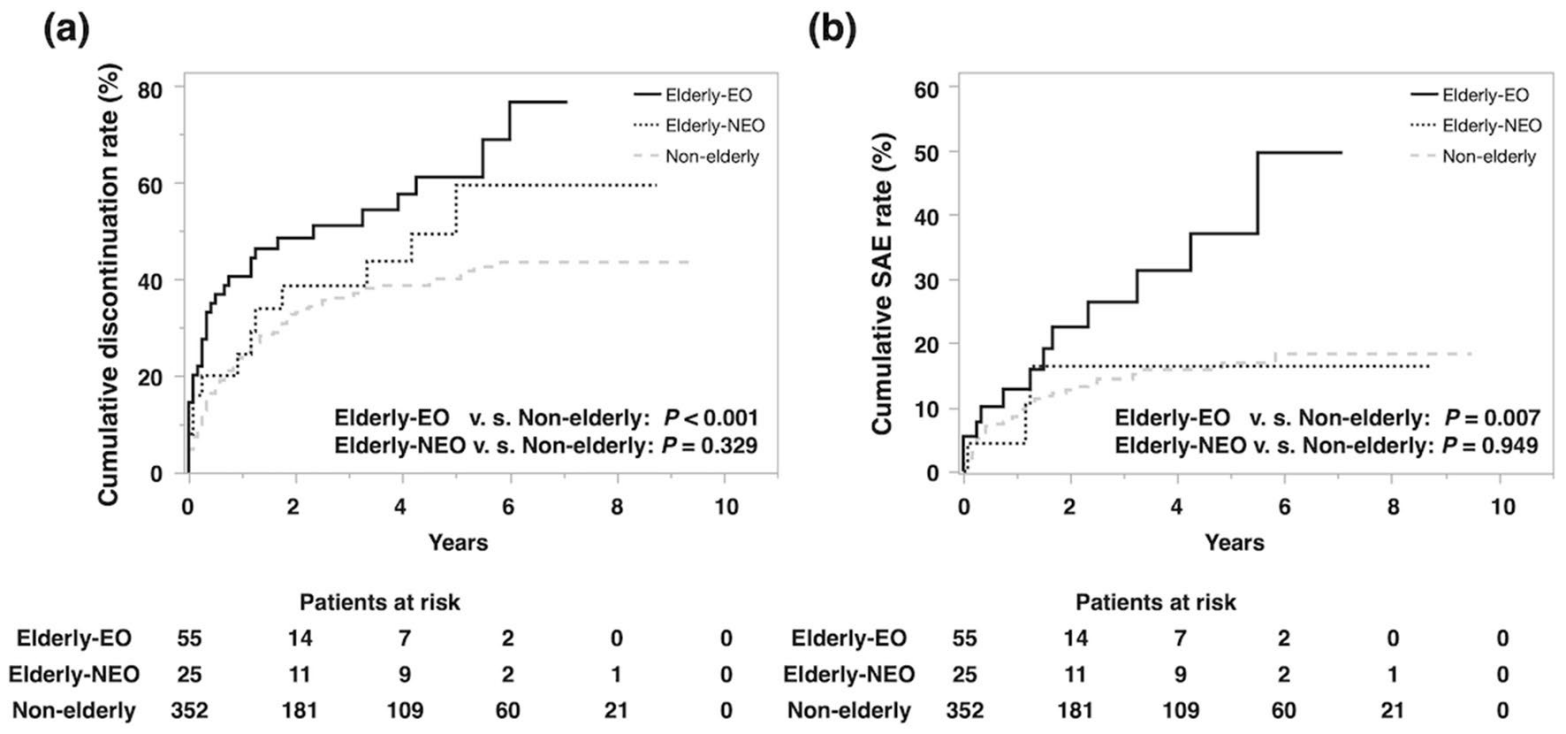
Cumulative events rate leading the discontinuation of anti-TNF treatment. (**a**) Cumulative discontinuation rate in Elderly-EO, Elderly-NEO, and Non-elderly. (**b**) Cumulative SAE rate during first anti-TNF treatment in Elderly-EO, Elderly-NEO, and Non-elderly. *Anti-TNF* anti-tumor necrosis factor, *EO* elderly onset, *IBD* inflammatory bowel disease, *NEO* non-elderly onset, *SAE* serious adverse event.

We further investigated the safety of anti-TNF treatment for IBD patients by evaluating the cumulative discontinuation rate and SAE rate of anti-TNF treatment. Although the cumulative discontinuation rate and SAE rate of anti-TNF treatment did not differ significantly between the Elderly-NEO and Non-elderly groups (*P* = 0.329 and *P* = 0.949, respectively), these were significantly higher in the Elderly-EO group than in the Non-elderly group (*P* < 0.001 and *P* = 0.007, Fig. 2a,b), In addition, we investigated the details of the SAEs by evaluating the incidence rate of individual SAEs normalized to 1000 py (Table 4). The incidence rates of infection (18/1000 py), cardiovascular event (9/1000 py), malignancy (18/1000 py), and death (27/1000 py) were higher in the Elderly-EO than in the Non-elderly groups (3/1000 py, 2/1000 py, 2/1000 py and 2/1000 py, respectively; Table 4). None of these factors, however, was significantly different between the Elderly-NEO and Non-elderly groups.

**Table 4:** The events leading the discontinuation of anti-TNF treatment.

Events	Non-elderly n = 352		Elderly			
			Elderly-NEO n = 25		Elderly-EO n = 55	
	n	n/1000 py	n	n/1000 py	n	n/1000 py
Lack of effectiveness	89	132	9	131	20	291
Primary non-response	25	40	3	44	9	164
Loss of response	64	92	6	87	11	127
SAEs	44	45	3	40	14	127
Infection	3	3	0	0	2	18
Infusion reaction	17	17	1	13	1	9
Exanthema	5	5	1	13	1	9
Lupus-like syndrome	7	7	0	0	1	9
Interstitial pneumonia	0	0	0	0	1	9
Thrombocytopenia	0	0	0	0	1	9
Hepatic dysfunction	2	2	1	13	0	0
Renal dysfunction	2	2	0	0	0	0
Cardiovascular event	2	2	0	0	1	9
Malignancy	2	2	0	0	2	18
Death	2	2	0	0	3	27
Others	3	3	0	0	1	9

*Anti-TNF* anti-tumor necrosis factor, *EO* elderly onset, *IBD* inflammatory bowel disease, *NEO* non-elderly onset, *py* patient-years, *SAEs* severe adverse events.

## Discussion

The present study evaluated the efficacy and safety of anti-TNF treatment in bio-naïve elderly patients with IBD according to age at onset (Elderly-EO and Elderly-NEO). The study was conducted in a multicenter setting including both an academic institution and 17 non-academic institutions and demonstrated, with standardized disease activity scores, the efficacy of anti-TNF treatment for bio-naïve Elderly-EO. The results clearly demonstrated that bio-naïve Elderly-EO IBD patients exhibit a decreased response to anti-TNF treatment compared with Non-elderly IBD patients, and that long-term efficacy of anti-TNF treatment for bio-naïve Elderly-EO could be expected if the IBD-type was CD, the disease duration was less than 1 year, and the patients were without concomitant corticosteroid treatment at the start of anti-TNF treatment. Furthermore, the results indicated that SAEs, including infection, cardiovascular events, malignancy, and death, were more frequent in Elderly-EO than in Elderly-NEO or Non-elderly IBD patients.

A few previous studies demonstrated the efficacy and safety of initial anti-TNF treatment for elderly patients with IBD^23–25^. These studies showed that elderly patients with IBD had a lower long-term continuation rate of initial anti-TNF treatment due to SAEs, not to a loss of response^24,25^, and a lower short-term response rate as judged by the physicians^23,24^, not according to standardized disease activity indexes as used in the present study. Here, we first demonstrated the short- and long-term clinical or steroid-free remission rates of elderly patients with IBD using pMayo and HBI scores, which are generally used as disease activity indexes for patients with IBD. Also, by dividing patients according to their age at disease onset, we revealed that the long-term effectiveness of anti-TNF treatment was lower in bio-naïve Elderly-EO patients, and the short- and long-term effectiveness of anti-TNF treatment in Elderly-NEO patients did not differ from those in Non-elderly patients. In addition, multivariate analysis showed that elderly onset was extracted as significant factors for both clinical and steroid-free remissions, whereas IBD type and concomitant corticosteroid were extracted only for each (Table 2). We then think that elderly onset was mainly associated with low efficacy of first anti-TNF treatment in patients with IBD rather than IBD type or concomitant corticosteroid. These data strongly suggest that anti-TNF treatment is less effective, especially for Elderly-EO patients with IBD, but further studies are required due to the small number of Elderly-NEO patients in the present study.

Patients with EO-rheumatoid arthritis (EO-RA) have higher serum interleukin-6 levels and lower serum TNFα levels than patients with NEO-RA, and high levels of TNFα are associated with higher risk of hospitalization and death^26–28^. Furthermore, anti-TNF treatment for EO-RA seems to be less effective than for NEO-RA^26^. Although serum and tissue drug concentrations of anti-TNF agents reflect long-term sustained clinical remission in CD^29^, anti-infliximab antibody levels are increased in elderly patients with IBD despite their immunosenescence^30^. No molecular studies to date have analyzed the effect of anti-TNF treatment in EO-IBD, and future studies to investigate the pharmacokinetics of anti-TNF treatment in EO-IBD are warranted.

By investigating the patient characteristics and concomitant drugs in Elderly-EO, we revealed that an IBD-type of CD, short disease duration, and no concomitant corticosteroid treatment are factors associated with the long-term efficacy of anti-TNF treatment for bio-naïve Elderly-EO patients. A previous report demonstrated that early corticosteroid exposure was a significant risk factor for IBD-related surgery in Elderly-EO^15^. Therefore, earlier transition to anti-TNF treatment is expected to have long-term efficacy for Elderly-EO patients with corticosteroid dependence or resistance. In addition, top-down treatment with anti-TNF agents or accelerating step up to anti-TNF treatment with determining the effect of corticosteroid with short disease duration have potential to be more effective for elderly onset patients with CD.

Some previous reports assessed the safety of anti-TNF treatment for elderly patients with IBD and demonstrated that older age was the only independent factor for SAEs, including infection, malignancy, and cardiovascular and death events^23–25,31^. Consistent with the previous reports, we revealed that SAEs leading to discontinuation of anti-TNF treatment were more frequent in Elderly-EO than in Non-elderly patients, and the cumulative SAE rate was significantly higher in Elderly-EO than in Non-elderly patients, especially at 2 years after the first anti-TNF treatment. In the present study, the proportion of concomitant immunomodulators in Elderly-EO was significantly higher than Non-elderly in the present study (Table 1). However, when analyzed in Elderly-EO, the proportion of SAE was unchanged between the concomitant immunomodulator group (31.8%) and the non-concomitant group (18.2%, *P* = 0.243). In the present study, we defined SAE as the events leading to the discontinuation of anti-TNF treatment and we didn’t evaluate adverse events that could be managed without discontinuing the anti-TNF treatment. From these, in Elderly-EO, careful and close follow-up is required in Elderly-EO patients to assess SAEs with or without using concomitant immunomodulators, even if the anti-TNF treatment is effective.

The present study has several limitations. First, because this study was a retrospective study, a selection bias for patients receiving anti-TNF treatment could not be excluded, accurate information on comorbidities could not be collected, and endoscopic evaluation could not be performed at a designated time. Second, multivariate analysis for factors associated with clinical or steroid-free remission after 52 weeks of anti-TNF treatment could not be conducted due to the small number of Elderly-EO patients. A large and prospective validation study is required to confirm the results of this study. Third, fecal calprotectin levels were not measured because it had not been approved by insurance for patients with CD in Japan. Forth, we could not assess the frailty which was reported to affect disease activity, and treatment efficacy and safety in elderly patients with IBD^32,33^. To address these issues, a prospective study with comorbidities, frailty, endoscopic and fecal evaluation is needed.

In conclusion, anti-TNF treatment for bio-naïve EO-IBD may be less effective and raise safety concerns.

## Methods

### Patients

This was a retrospective multicenter study. Patients at least 16 years of age that were diagnosed and hospitalized with IBD, including UC or CD, and started treatment with biologic agents (infliximab, adalimumab, golimumab, ustekinumab, or vedolizumab), tofacitinib, or tacrolimus from January 2010 to March 2019 due to corticosteroid dependence or resistance at 18 hospitals participating in the Osaka Gut Forum were enrolled in the study in November 2019. Of them, bio-naïve patients with IBD who underwent anti-TNF treatment were included in the analysis. Patients who had undergone a total colectomy and/or ostomy before the treatment were excluded. The study was carried out in accordance with the Declaration of Helsinki, and approved by the ethics committee of Osaka University Hospital and the other ethics committees (Supplemental Table 2). Written informed consent was waived by ethics committee of Osaka University Hospital, and the other ethics committees (Supplemental Table 2), by giving participants the opportunity to opt out.

### Definition of elderly and patient grouping

According to previous reports^5–7^, we defined elderly patients as those 60 years of age or older, and EO as patients who were at least 60 years of age at the time of diagnosis. Furthermore, elderly patients with IBD were further divided into those with elderly onset (Elderly-EO) and those with non-elderly onset (Elderly-NEO).

### Data collection

For this study, patient characteristics (sex; age at the start of treatment with biologic agents, tofacitinib, or tacrolimus; age at disease onset; disease duration; IBD-type [UC or CD]; disease phenotype according to the Montreal classification^34^; smoking status [smoking or past smoking, or never]; prior IBD surgery; anti-TNF agents [infliximab, adalimumab or golimumab], ustekinumab, vedolizumab, tofacitinib, tacrolimus, concomitant IBD medication [5-aminosalicylic acid, corticosteroid, immunomodulators]; clinical features and blood test [C-reactive protein, albumin] at week 0 of anti-TNF treatment; clinical features at 8 [+−4] weeks and 52 [+−8] weeks of anti-TNF treatment; and reasons for treatment discontinuation) were extracted from medical records at each institution. Clinical features were obtained by a partial Mayo score (pMayo) for UC^35^ and the Harvey-Bradshaw index (HBI) for CD^36,37^. Lack of effectiveness was defined as either primary non-response or loss of response, resulting in a switch of therapy or surgery^25^. Severe adverse events (SAEs) were defined as infection, infusion reaction, exanthema, lupus-like syndrome, interstitial pneumonia, thrombocytopenia, hepatic dysfunction, renal dysfunction, cardiovascular events, malignancy, death, and others leading to the discontinuation of anti-TNF treatment.

### Outcomes

Efficacy of the anti-TNF treatment was assessed by the clinical remission and steroid-free remission rates at 8 weeks and 52 weeks of anti-TNF treatment. In addition, factors associated with the clinical remission and steroid-free remission rates were assessed at 8 and 52 weeks of anti-TNF treatment. Clinical remission was defined pMayo score of ≤ 2 and no individual sub-score > 1 in UC^38^, and an HBI score of ≤ 4 in CD. To evaluate safety, we calculated the cumulative SAE rate during anti-TNF treatment from the start of anti-TNF treatment to the cessation of treatment or follow-up and individual SAE rate during anti-TNF treatment as the number of SAEs divided by 1000 patient-years [(py) i.e., events per 1000 py].

### Statistical analysis

Continuous variables are presented as the medians and interquartile range (IQR). Categorical valuables are presented as frequencies. Differences in the distribution of variables were evaluated using Pearson’s chi square test or Fisher’s exact test if the numbers were smaller than 5. We used Bonferroni’s method for multiple comparisons (Elderly-EO to Non-elderly or Elderly-NEO to Non-elderly) and a significant *P* value was defined as *P* < 0.05/2 = 0.025. The odds ratio (OR) and corresponding 95% confidence interval (CI) were estimated by multivariate logistic regression analysis with the stratification variables. The cumulative incidence rate was estimated by the Kaplan–Meier method and evaluated by the log-rank test. In these evaluations, *P* values less than 0.05 were considered significant. Statistical analyses were performed using JMP statistical software (version 16.0.0; SAS Institute, Inc., Cary, NC, USA).

### Ethnics approval and consent to participate

The study was carried out in accordance with the Declaration of Helsinki, and approved by the ethics committee of Osaka University Hospital and the other ethics committees (Supplemental Table 2). Written informed consent was waived by ethics committee of Osaka University Hospital, and the other ethics committees (Supplemental Table 2), by giving participants the opportunity to opt out.

### Consent for publication

This study contains no individual person’s data in any form.

## Supplementary Information

Supplementary Information.

## Data Availability

The datasets generated and/or analyzed during the present study are available from the corresponding author upon reasonable request.
