# Supplementary material for: Elderly onset age is associated with low efficacy of first anti-tumor necrosis factor treatment in patients with inflammatory bowel disease
Source: Sci Rep. 2022 Mar 29;12:5324. doi: 10.1038/s41598-022-09455-8 (PMC8964802; doi:10.1038/s41598-022-09455-8)
Supplement: Supplementary file 1 — Supplementary Information. [file 41598_2022_9455_MOESM1_ESM.docx]

**Supplemental Table 1.** Multivariate analysis of factors for clinical remission or steroid-free remission after 8 weeks of anti-TNF treatment (n= 380).

|  | Clinical remission | | Steroid-free remission | |
| --- | --- | --- | --- | --- |
|  | OR (95% CI) | *P* value | OR (95% CI) | *P* value |
| Age at onset (EO) | 0.77 (0.39-1.52) | 0.462 | 0.61 (0.29-1.24) | 0.177 |
| Sex (Male) | 1.07 (0.64-1.79) | 0.775 | 0.78 (0.55-1.56) | 0.781 |
| Disease duration> 1 year | 0.58 (0.33-1.01) | 0.057 | 0.61 (0.35-1.07) | 0.088 |
| Current or past smoking | 0.79 (0.46-1.35) | 0.405 | 0.87 (0.50-1.49) | 0.616 |
| IBD-type (UC) | 0.27 (0.14-0.52) | < 0.001 | 0.53 (0.28-1.01) | 0.055 |
| CRP <0.30 mg/dl | 1.93 (1.10-3.39) | 0.020 | 2.31 (1.30-4.08) | 0.003 |
| Alb <3.6 g/dl | 0.86 (0.50-1.48) | 0.599 | 1.33 (0.77-2.29) | 0.296 |
| Anti-TNF agents |  |  |  |  |
| Infliximab | 1.0 (reference) |  | 1.0 (reference) |  |
| Adalimumab | 1.14 (0.68-1.91) | 0.602 | 1.34 (0.80-2.25) | 0.255 |
| Golimumab | 1.90 (0.68-5.25) | 0.215 | 0.97 (0.34-2.76) | 0.967 |
| Concomitant drugs |  |  |  |  |
| Corticosteroid | 0.80 (0.45-1.44) | 0.470 | 0.22 (0.12-0.40) | < 0.001 |
| Immunomodulators | 0.71 (0.40-1.23) | 0.228 | 0.75 (0.42-1.35) | 0.350 |

Alb: albumin, Anti-TNF: anti-tumor necrosis factor, CI: confidence interval, CRP: C-reactive protein, EO: elderly onset, IBD: inflammatory bowel disease, OR: odds ratio, UC: ulcerative colitis

**Supplemental Table 2.** The List of ethics committees that approved the study and waived informed consent by giving participants the opportunity to opt out.

| No. | The name of ethics committee |
| --- | --- |
| 1 | The ethics committee of Osaka University Hospital |
| 2 | The ethics committee of Osaka Rosai Hospital |
| 3 | The ethics committee of National Hospital Organization Osaka National Hospital |
| 4 | The ethics committee of Toyonaka Municipal Hospital |
| 5 | The ethics committee of Osaka General Medical Center |
| 6 | The ethics committee of Osaka Police Hospital |
| 7 | The ethics committee of National Hospital Organization Osaka Minami Medical Center |
| 8 | The ethics committee of Kansai Rosai Hospital |
| 9 | The ethics committee of Ikeda City Hospital |
| 10 | The ethics committee of Itami City Hospital |
| 11 | The ethics committee of Higashiosaka City General Hospital |
| 12 | The ethics committee of Otemae Hospital |
| 13 | The ethics committee of Nishinomiya Municipal Central Hospital |
| 14 | The ethics committee of Japan Community Healthcare Organization Osaka Hospital |
| 15 | The ethics committee of NTT-West Osaka Hospital |
| 16 | The ethics committee of Hyogo Prefectural Nishinomiya Hospital |
| 17 | The ethics committee of Saiseikai Senri Hospital |
| 18 | The ethics committee of Yao Municipal Hospital |
